# Upward Feedback: Exploring Learner Perspectives on Giving Feedback to their Teachers

**DOI:** 10.5334/pme.818

**Published:** 2023-03-22

**Authors:** Katherine Wisener, Kimberlee Hart, Erik Driessen, Cary Cuncic, Kiran Veerapen, Kevin Eva

**Affiliations:** 1 Faculty Development, Faculty of Medicine, University of British Columbia, Canada; 2 School of Health Professions Education (SHE), Maastricht University, Maastricht, the Netherlands; 3 Faculty of Medicine, University of British Columbia, Canada; 4 Department of Educational Development and Research, Faculty of Health Medicine & Life Sciences, Maastricht University, Maastricht, the Netherlands; 5 Division of General Internal Medicine, University of British Columbia, Vancouver. BC, Canada; 6 Department of Medicine, University of British Columbia, Vancouver, BC, Canada

## Abstract

**Introduction:**

Feedback from learners is known to be an important motivator for medical teachers, but it can be de-motivating if delivered poorly, leaving teachers frustrated and uncertain. Research has identified challenges learners face in providing upward feedback, but has not explored how challenges influence learners’ goals and approaches to giving feedback. This study explored learner perspectives on providing feedback to teachers to advance understanding of how to optimize upward feedback quality.

**Methods:**

We conducted semi-structured interviews with 16 learners from the MD program at the University of British Columbia. Applying an interpretive description methodology, interviews continued until data sufficiency was achieved. Iterative analysis accounted for general trends across seniority, site of training, age and gender as well as individual variations.

**Findings:**

Learners articulated well-intentioned goals in relation to upward feedback (e.g., to encourage effective teaching practices). However, conflicting priorities such as protecting one’s image created tensions leading to feedback that was discordant with teaching quality. Several factors, including the number of feedback requests learners face and whether learners think their feedback is meaningful mediated the extent to which upward feedback goals or competing goals were enacted.

**Discussion:**

Our findings offer a nuanced understanding of the complexities that influence learners’ approaches to upward feedback when challenges arise. In particular, goal conflicts make it difficult for learners to contribute to teacher support through upward feedback. Efforts to encourage the quality of upward feedback should begin with reducing competition between goals by addressing factors that mediate goal prioritization.

## Introduction

In the health professions, feedback from learners has been reported as a powerful motivator of clinicians’ commitment to teaching [1,2]. That said, teachers also report receiving brief, vague and generic feedback from learners, oftentimes months after a given teaching encounter, which can leave them feeling frustrated and uncertain regarding interpretation and application [3,4,5,6]. While research has highlighted challenges learners face in providing feedback to their teachers, we lack a fulsome understanding of how these challenges impact learners’ approaches towards upward feedback, why these challenges lead learners to resort to vague or generic feedback, and which teachers might be most susceptible to receiving suboptimal feedback. Exploring learners’ perspectives on giving feedback to their teachers to gain clarity regarding how and what learners offer to teachers can guide efforts to reduce the risks inherent in misleading or suboptimal feedback.

### Background

A learner providing feedback to their teacher is an instance of “upward feedback”, a term originating from organizational psychology [3,7,8]. Whether it be in-person, verbal conversations after a clinical learning encounter, anonymized student evaluations of teaching (SET) [9] after classroom-based education, or otherwise, upward feedback offers learners a voice in how teaching could be improved. Such opportunities for teachers to receive guidance on their performance are critical to increase the overall quality of health professions education [10].

That said, research within medical education and the broader field of education have highlighted common challenges learners face when assessing or giving feedback to their teachers: 1) having to complete too many forms that are time consuming [5]; 2) feeling ill-equipped to provide feedback to teachers [3,6,11]; 3) feeling uncertain about teachers’ receptivity and the impact of their feedback on teaching practices [6,11,12,13]; and, 4) experiencing power imbalances and fearing consequence [3,11,12]. These issues have contributed to a situation in which concerns regarding the validity and utility of SETs are widespread [14,15,16], making it likely that the value of narratives from learners [17] is all too easily overlooked.

These challenges offer a starting point for where health professions programs and teachers should focus efforts to enable learners to offer meaningful feedback. For example, remaining mindful of the number of times learners are asked to provide upward feedback, offering training on how to do so, and reinforcing to learners that their feedback is important appear to be worthwhile strategies and all seem fairly straightforward. As is often the case, however, addressing challenges may be more complex than first impressions would suggest. Efforts to address power imbalances, for example, have led to anonymized teacher assessment, but learners *still* report choosing wording that is vague and indirect due to concern their feedback is not truly anonymous [11]. Anonymization also leads to other challenges, namely, the delayed and decontextualized feedback that causes frustration for teachers [3,9,12,18].

In general, such lists of challenges, therefore, offer a necessary but not sufficient framework for thinking about how to improve upward feedback processes. To move forward we require a more nuanced understanding from learners themselves regarding how upward feedback challenges influence their goals for giving feedback to teachers, under which conditions challenges may be particularly prominent, and which factors may alleviate challenges in certain contexts. To that end, we sought to explore the following: 1) what are medical learners’ goals, approaches, and considerations in providing feedback to their teachers?; and, 2) how do learners navigate barriers in providing upward feedback when they arise?

## Methods

### Study Design

We applied an interpretive description methodology to explore learner perspectives about upward feedback [19]. Interpretive description originated in the field of nursing and is gaining traction more broadly in health professions education because it is meant to enable practice improvement [20]. Its theoretical underpinnings follow constructivism, with aims to capture multiple subjective experiences within a population by identifying patterns and generating rich descriptions [20]. Its applied nature aligns with our purpose, to enable improvements in learners’ provision of upward feedback. Ethics approval was obtained from UBC’s Behavioural Research Ethics Board (H20-04040).

### Setting

Our study took place within the MD program at the University of British Columbia (UBC) which spans four-years and four regional campuses. While each campus has its own context influenced by size, geographic location, and culture, learners progress through a standardized curriculum [21]. With respect to formal program evaluation, learners are asked to complete a series of Likert-scale questions about teaching and the learning environment, to write both positive and constructive narrative feedback, and to provide an overall score for each teacher at the end of each learning session. Additionally, end of rotation bi-directional feedback discussions are expected and learners sometimes opt to undertake informal feedback processes (e.g., cards and gifts). For the sake of this study, we treated ‘upward feedback’ as any effort learners reported as a means of conveying information about teaching performance to their educators.

### Participants

Medical students were recruited via email and an invitation posted to a social media group and were offered a $25 gift card. A subset of those were interviewed with selection of participants being purposive (i.e., effort was made to maximize variability by recruiting from the full continuum of seniority, across geographically distinct campuses, and by being inclusive of participants’ ages and genders). Such purposive sampling enabled a rich understanding of how upward feedback approaches varied in different contexts, enabling theoretical and practical implications to be drawn that apply to a range of settings.

### Data Collection

A semi-structured interview guide was developed by KW (a PhD candidate and faculty developer) and KE (medical education researcher) with questions constructed to capture learners’ perspectives on their role and activity as feedback providers, including motivators and facilitators, barriers and challenges. The interview guide was piloted with KH (a medical student co-investigator) to ensure the questions would resonate with learners and yield meaningful answers. All interviews were subsequently conducted through Zoom by KW and/or KH. All participants were unknown to the interviewers except in two instances of KH interviewing classmates. Interviews began with an invitation to explore how learners approached telling their teachers what was (and was not) valued about their learning experience. We did not provide a specific definition of upward feedback, and used the phrase ‘feedback to teachers’ during the interviews to let participants decide what they wanted to bring forward. The guide underwent iterative changes when analysis indicated a need for more targeted exploration. For example, while we initially explored learners’ approaches to giving feedback to strong teachers relative to weaker teachers, it became clear we also needed to include ‘average’ teachers as learners’ approaches varied across the full continuum. As recommended by the developers of interpretive description methodology, recruitment stopped after data sufficiency was reached, meaning that we ended recruitment when the full team of investigators, all of whom are “thoughtful educators” (to paraphrase Thorne, Kirkham & O’Flynn-Magee), agreed that the findings were plausible and that the analysis appeared to tell the full story participants were able to provide and adequately enabled practical advice to be delivered [22]. See Appendix A for the final interview guide.

### Analysis

Again, following the interpretive description technique, we undertook iterative phases of collecting, reviewing, and interpreting data, creating codes that were synthesized into patterns and relationships, and always remaining sensitive to how our observations might be applied in practice.

All audio recordings were listened to with analytic memos made and reviewed by KH and KW. Both interviewers debriefed about their observations after early interviews. Memos and an audit trail were kept to help the researchers engage in reflexivity and make an effort to separate participant sentiments from investigators’ expectations. To the same end, early transcripts that offered contrasting perspectives were reviewed by three investigators (KW, KH, and KE) who subsequently discussed their observations. KW drafted a codebook combining all three researchers’ impressions, which was continuously expanded upon as additional transcripts revealed new codes. The full group of investigators then iteratively reviewed additional transcripts, applied the codebook, discussed their interpretations, refined and grouped codes to reflect broader themes, and met to discuss their interpretations and code assignments. Throughout this process, investigators who primarily identify as medical education researchers (KE, ED), faculty developers (KW, KV), a clinician (CC) and a medical learner (KH) were sensitive to and explicit about to how their own backgrounds were influencing their perspectives of the data. After a series of cycles, operational definitions were generated for all codes and illustrative quotes were highlighted. The final codebook is in Appendix B.

## Results

The invitation to participate received an unusually strong response with over 100 learners volunteering. Sixteen were ultimately included in the study before data sufficiency was concluded. Participant demographics are summarized in Table 1.

**Table 1:** Participant Demographics.


SITE OF TRAINING	YEAR OF STUDY	AGE RANGE	GENDER

Central: 6 Distributed: 9	Year 1: 3	20–29: 11	Female: 11

	Year 2: 1	30–39: 2	Male: 3

	Year 3: 4	40–49: 2	Non-binary: 1

	Year 4: 7		


**Figure 1:**
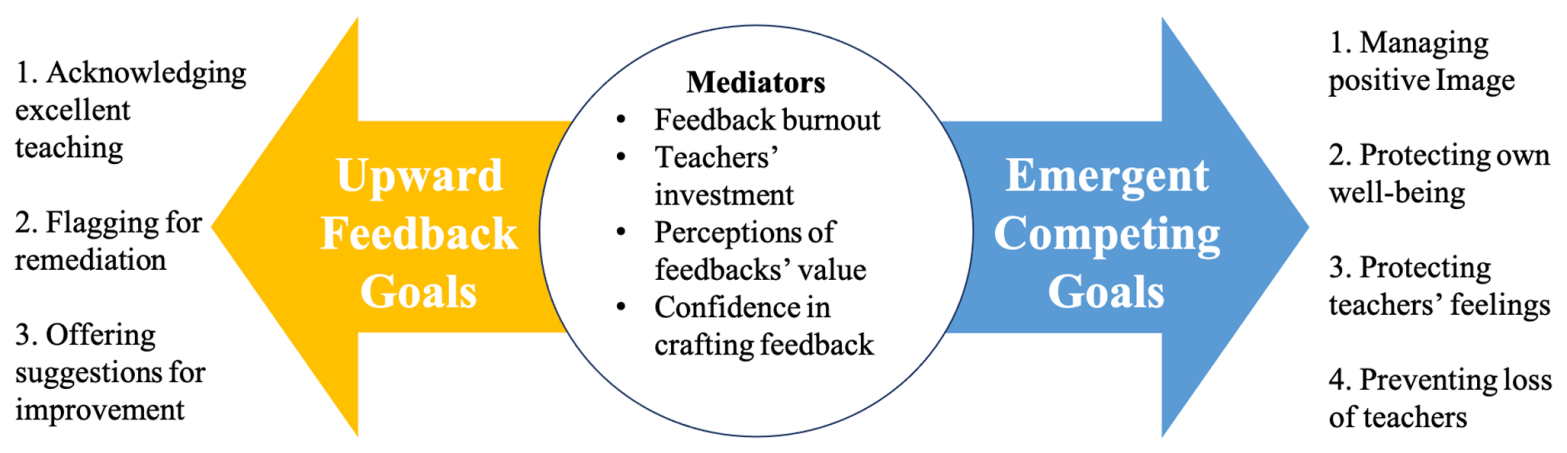
Tensions between Learners’ Goals in Providing Upward Feedback.

Interviews ranged from 21 to 75 minutes. Provision of upward feedback appeared to be an important issue for learners as participants demonstrated considerable thoughtfulness and deliberation, even becoming emotional at times when recounting teaching experiences that influenced their approach to offering feedback. As learners shared their experiences, they tended to articulate a variety of well-intentioned goals while also noting competing aims that created tension and sometimes prevented or altered upward feedback efforts. Figure 1 offers a visual graphic of the complexities that are described below.

### Upward Feedback Goals and Approaches

Many learners were keen to provide positive feedback to express the extent to which they valued excellent teaching, while constructive or critical feedback was reported to be a means to improve negative learning experiences for both themselves and future learners. Some learners sought to achieve these goals by relying solely on formal processes (namely, through teacher assessment forms). Others exercised a greater sense of agency and used a multitude of approaches such as waiting in line after a teaching encounter to offer feedback, purchasing personal gifts or cards, nominating teachers for awards, etc. Importantly, several learners reported that they considered indirect expressions of participation to be a means of delivering feedback, which included purposefully engaging in a learning encounter by asking questions and acting invested, or conversely, withdrawing participation if the encounter was not going well:

*…I think of it kind of is like a reward almost. Like you know you get what you put in so I think if [the teacher] is putting in that effort and that time and you know a session that’s quite engaging and well done and organized and you know up to the standards that I think are a good session then they’re gonna get all of that in return. Um, and so I think it does provide good feedback*. (P3)

When learners had goals of improving teaching quality, they generally opted to provide critical feedback anonymously or to program leadership instead of directly delivering it to teachers although some initiated these difficult conversations in-person, feeling a sense of duty to speak up on behalf of their peers:

*I feel like as a person who has a lot of confidence I know when people wouldn’t speak up…and if I can have some impact on ending some cycle or at least, you know, that’s something I’m like willing to do*. (P4)

### Emergent Competing Goals

While learners adopted a variety of formal and informal feedback approaches and modalities that varied depending on their specific goals (i.e., to improve teaching quality, express gratitude, flag problematic teachers, etc.), they also engaged in a goal-weighing process as they expressed additional underlying and conflicting priorities. Maintaining a positive image in the eyes of their teachers, protecting their own well-being, preventing teachers’ feelings or teaching contracts from being negatively impacted by critical feedback were, therefore, considered competing goals that led to tensions that impacted the feedback they offered.

Some tensions were straightforward as learners weighed the possibility that negative upward feedback might have a detrimental impact on their career. This was particularly relevant to a few learners who self-identified as racialized or otherwise marginalized (i.e., those who were markedly older than their peers or who were not heteronormative). Others recounted times they offered undeservedly positive feedback or gifts to teachers because of the potential goodwill that might afford. Learners were, however, highly sensitive to the risk inherent in efforts to manage such tensions. For example, regardless of stage of training, participants often hesitated to give positive feedback for fear of seeming overly eager, sentimental, or unprofessional. In such cases, learners preferred not to subject themselves to additional judgements, so said only what was required by the learning experience:

*I feel like they would be scrutinizing or judging the words or that because I’m so down the ladder my words don’t mean that much to them so I don’t want to take up too much of their time or I don’t want to seem overly eager or overly sentimental or anything like that…like if I ended the rotation good, and I knew the [preceptor] saw me in positive light, I didn’t want to say anything that would change that…* (P8)

Similarly, while participants articulated a desire to improve quality of teaching, they often spoke of how offering meaningful feedback to every teacher was time consuming to the point that it sacrificed their well-being, which created tension by reducing learners’ capacity to fulfil that goal:

*…I think probably a combination of just me being kind of burnt out and overwhelmed by my responsibilities…I just figured like oh I guess I’ll write something [generic] for them* (P15).

This too, however, was not simply a matter of giving or not giving feedback. Rather, learners reported feeling forced to pick and choose to whom to offer feedback in a way that often resolved by prioritizing those who made an extreme positive or negative impact. As a result, the majority of teachers (i.e., those who were perceived as ‘average’) were least likely to receive feedback because constructing unique content for those who do not stand out took more time and effort:

*In terms of providing feedback, I think it’s pretty tough when you have something that’s average cause there’s not really, you don’t really have anything to critique per se but you don’t really have any overwhelmingly good experiences either*. (P10)

Rather than being sensitive only to their own experiences, learners showed an awareness of others in hesitating to offer feedback if they felt it could hurt their teachers’ feelings or de-motivate those who were known to be in limited supply to the medical education program (e.g., specialists who are few in number). They acknowledged and empathized with the notion that barriers sometimes prevented clinicians from prioritizing teaching and tended to abort giving feedback in such circumstances:

*…it was almost like they were so positive about like our group, this was for [Case-Based Learning], um, and how things were going that I didn’t want to like hurt them almost or like break that…*(P11)

That was true especially if an observed issue wasn’t considered critical. Even when teaching quality was perceived as poor or bordering on mistreatment, however, they would hold back for fear that feedback could result in the removal of a needed teacher.

*…[if] all of my concerns are validated and like they decide to kind of remove one teacher, there would be a huge loss to future students who may not be able to get as much exposure* (P13)

In sum, while learners may have earnest goals with respect to upward feedback, competing priorities created barriers that resulted in tensions that could lead learners towards providing vague feedback, reducing effort, or avoiding upward feedback altogether. Whether or not competing goals took precedence and led to suboptimal feedback, however, was determined by various factors that mediated the extent of goal conflict.

### Factors Mediating which Goal takes Priority

When upward feedback goals came into conflict with emergent competing goals, various factors mediated which goal took precedence for learners. Specifically, manageable amounts of upward feedback requests, teachers who were interested in learners’ feedback, perceptions that feedback was valued, and feeling confident in crafting feedback created conditions for learners to invest effort into upward feedback practices in earnest. For example, learners reported being more likely to resort to brief and generic commentary (abandoning their more constructive goals) when they felt particularly exhausted from feedback requests. Upward feedback fatigue mediated the extent to which protecting one’s well-being outweighed their desire to help teachers improve:

*…it just feels, like the more you have the less motivated I am cause it just feels like a bigger burden to go in and fill them all out*. (P9)

Motivation to power through all feedback requests varied as reflected in the preceding quote, as did learners’ willingness to offer constructive feedback to their teachers. Whether learners took the risk of negatively impacting their own image, or hurting their teachers’ feelings, was mediated by the extent to which a teacher explicitly and genuinely invited feedback from learners. When teachers were perceived as not being invested, or when requests for feedback appeared superficial, learners felt justified in their decision to not spend the time required or take the risk inherent in delivering truthful feedback:

*I think that, uh, since students are so worried about power dynamics and you know knowing that we’re being evaluated is a huge barrier to providing honest and negative feedback and so if preceptors specifically ask for negative feedback, then they’re much more likely to receive it…*. (P12)

This weighing process could itself be effortful as, for example, gauging a teacher’s receptivity to feedback was challenging when learners worked with preceptors for a short period of time. Time together was not, however, the ultimate determinant of connecting with learners as many reported instances in which receptivity was effectively established during a brief learning encounter through an explicit and genuine expression of interest while others found that a sufficient relationship was not built even over a longitudinal experience.

Gauging the extent to which learners felt feedback had an actual impact on teachers’ behaviour was another common mediator. Witnessing or hearing about how feedback has been applied by teachers led learners to want to contribute to teacher support through upward feedback. On the other hand, perceptions that feedback was not taken seriously by the teacher or medical school led to a sense of distrust in the feedback process. In these cases, learners withheld their feedback to avoid feeling as though their troubling experiences with some teachers weren’t valued, thus preserving their own well-being. The cynicism exhibited by some participants was particularly troublesome, as a sense of futility applied to giving feedback to all teachers, not simply the problematic ones:

*… I talked to other students about this preceptor, senior students, and they’d worked with her and they’d had the same issues. So it feels like well the school knows about this so like if they know about it and they’re putting me in this situation then it’s my problem. Nothing I’m gonna say is gonna change this so why would I bother… I don’t know it just feels like things don’t change*. (P1)

Whether a learner had the confidence to make judgements about the quality of teaching or to craft feedback effectively also mediated the extent to which goals of giving meaningful feedback to teachers won out over alternative priorities. When learners lacked confidence, they were more likely to internalize suboptimal teaching experiences as being their own fault rather than opportunities to help the teacher improve:

*… I was very not confident in my own ability as a [learner], so I internalized a lot of like the negative events occurring and genuinely felt like I just wasn’t doing well so it was fair to have staff like maybe not react very well or not be very supportive because I just felt like I was a burden. Um, but kind of later on in my rotations I developed a bit more confidence in my skill and ability, that’s when I felt like when I had a negative situation I was able to tell myself that this is not me, this is the situation or this is not me, this is the preceptor*. (P7)

Learners not only grappled with knowing whether to say something, but also with knowing how to say it in a way that would not harm the teacher’s feelings or teaching position:

*I worry that, um, the tone, you know, it might not come through that I’m actually trying to offer constructive feedback. Um, yeah and all those other things, like I don’t want them to say ‘oh I’m not a good teacher’ and then stop teaching because we need teachers*. (P5)

## Discussion

Upward feedback is an important topic for learners, as illustrated by the high number of willing participants and the thoughtfulness with which learners recounted their experience and approaches. Anecdotal criticisms of learners not being interested or committed enough to provide feedback, that is, were discounted by the earnestness with which learners expressed desire to engage in upward feedback to improve the quality of teaching, to motivate teachers to continue contributing to medical education, and to flag problematic teacher behaviours to medical school leadership. That was reinforced by the variety of ways in which learners expressed taking steps to achieve such goals.

We also heard, however, about many factors that interfere with the actualization of upward feedback goals, making their provision anything but simple. Thus, in answer to our second research question, how do learners navigate barriers in providing upward feedback when they arise, we conclude that they do so through a goal weighing process (see Figure 1). While previous research has highlighted specific barriers learners face in providing upward feedback, our efforts expand on this knowledge base by representing challenges not as one-dimensional barriers with easy solutions, but rather as an intertwined set of internal conflicts and tensions that influence learners’ approaches. Which of many competing goals takes precedence was mediated by additional considerations that, in some cases, enabled learners to support teachers with meaningful feedback and, in others, led them to resort to vague, brief, or dishonest feedback. Such mediators, furthermore, could accumulate to the point of self-fulfilling prophecy. For example, a learner who felt their feedback was not valued was more likely to offer vague and brief feedback, which reduces teachers’ capacity to make any adjustments in response, thus reinforcing learners’ perceptions that feedback is not impactful or that their teachers are not receptive [23].

The literature on goal conflict offers valuable framing for these observations by differentiating between inherent conflicts (i.e., when pursuing one goal coincides with losing traction on another) and resource conflicts (i.e., when there is a lack of time or energy to pursue two separate goals) [24]. Learners’ descriptions of the clash between upward feedback goals and competing goals generally resembled inherent conflicts. For example, encouraging teachers with positive feedback was felt to counter one’s desire to appear competent and professional. Similarly, offering suggestions for improvement countered learners’ desire to not unduly harm teachers’ feelings or motivations to teach. With both types of conflict, an individual is pressured to choose one goal and abandon the other. That is important because inherent conflicts have been reported as posing particularly high risk of goal disengagement as a means to reduce the strong ambivalence felt from having two goals at odds with each other, thereby restoring psychological well-being [25].

Thus, a key implication from our study is the importance of recognizing that suboptimal feedback is not necessarily a simple matter of disinterest on the part of learners, but may reflect beneficial adaptations from the learner’s perspective (e.g., a self-protective act arising in response to competing goals). Viewing vague or absent feedback as an indication that a learner is grappling with conflicting goals may offer a valuable re-framing of the problem that offers a starting point for thinking about how to enable better upward feedback.

Digging deeper into such practical implications, it is worth acknowledging that inherent goal conflicts are particularly challenging to address in this instance given that they are likely, to a degree, a reflection of the social power and hierarchy ingrained in the culture of medical education where teachers are also potential future employers or, at the very least, able to influence the professional and educational outcome of learners. For example, regardless of the extent of effort one might engage to reduce threats, learners cannot rationally be counseled to ignore risks inherent in upward feedback given that medical teachers have openly admitted that feedback received affects how they assess learners [3]. Tackling these insidious dynamics requires a close examination of the ways in which social power limits learners’ perceived ability to communicate openly and honestly about their learning experiences [26]. Furthermore, it is perfectly human to worry about hurting teachers’ feelings and motivations given the recognition that clinicians are often volunteering their time to teach. Educational programs do not have infinite choice or control when it comes to filling teaching responsibilities and sometimes must retain “good enough” teachers or those who teach by virtue of where they work as opposed to those who have a genuine interest in teaching, which could fuel learners’ perspectives that their feedback isn’t acted upon [27].

One way to begin to address the complexities inherent in learners’ goal conflict is to think about whether efforts can be made to reduce the competition between goals by targeting the mediating factors. At a program level, it is worth scrutinizing motivations and approaches to ensure meaningful data are being prioritized over quantity of data. For example, recruiting a representative sample of learners to assess lectures rather than an entire class, may lessen the fatigue that derives from having too many feedback requests, thereby increasing the wherewithal learners have to offer thoughtful feedback. Similarly, supporting faculty to convey a genuine investment in their learners’ feedback could lead learners to push through competing priorities. This could be achieved not only by explicitly inviting learner feedback, but also by role modeling how constructive feedback can be delivered effectively, encouraging learners to engage in bidirectional feedback conversations to reduce the extent to which they feel paralyzed by the risk that their feedback is unwanted or hurtful. At the learner level, providing learners with examples of how leadership enacts remediation (e.g., faculty development) and recognition (e.g., promotions and awards) might help to ground the actual influence of upward feedback and lower the likelihood that learners avoid giving it. This is particularly important given that learners in this study both under- and over-estimated the power of their feedback as some felt their feedback never sees the light of day while others worried that it could interfere with teachers’ contracts. Lastly, while countless resources have been offered to faculty worldwide to help them support learners as recipients of feedback, rarely have tables been flipped by offering students a guide and concrete examples of how to provide effective feedback to their teachers.

Of this list of potential interventions, we recommend prioritizing those that have a focus on improving teacher-learner relationships (e.g., teachers investing in learners, role modeling effective feedback and explaining the impact of learner feedback) as these align with social exchange theory [28], which is what organizational behaviourists have leveraged to improve upward feedback processes. Social exchange theory posits that if learners perceive high quality relationships with their teachers this will lead to a sense of commitment and loyalty that may compel them to commit to delivering quality upward feedback [11].

In any case, addressing the specific factors that mediate which goals are given more weight might reduce the extent to which goals are seen as competing against another, thereby enabling learners to make progress towards all of their goals simultaneously (e.g., reducing upward feedback fatigue enables learners to provide quality upward feedback and maintain their well-being). Such efforts could go a long way to not only improving the quality of upward feedback, but also encourage the continuous quality improvement mentality that we expect learners themselves to adopt when they become practitioners [29].

When contemplating any intervention, the self-fulfilling prophesy mentioned earlier leads us to recommend that efforts be enacted early and reinforced throughout a medical learner’s education to avoid the vicious cycles that can emerge from an initial sense of skepticism. None of these strategies offer simple solutions, but when embedded in a genuine effort to nurture a learning culture, they offer promising leads built on learner perspectives.

### Limitations

Given that our study was conducted at a single institution in an undergraduate medical program, there is some risk that our findings lack transferability to other contexts. We attempted to mitigate that risk by exploring learners’ perspectives on upward feedback in a breadth of teaching contexts and through efforts to maximize the variation in our sample. Furthermore, our study design does not allow juxtaposition between learners’ views and those of the faculty to whom upward feedback is provided; nor did we differentiate how learners’ approaches varied based on characteristics of their teacher such as whether the teacher was a resident, an interprofessional team member, or an attending. Given the plausible influence of power and hierarchy, this could be an important area for future exploration. It is also likely that the lead authors’ previous experience working in faculty support/development coloured her interpretation of the observations made, but we guarded against being unduly influenced in this way by involving a diverse authorship group with varied roles in medical education, including a medical student, in data analysis.

## Conclusion

This study illustrates that learners have specific goals in engaging with upward feedback, which are countered by other competing goals; ultimately, how goals are enacted is determined by mediating factors that guide their prioritization. The complexities uncovered illustrate that vague or absent feedback is not a simple matter of learners being worried about repercussions or shirking a professional obligation. Rather, what poses threats to the delivery and veracity of upward feedback relates to issues inherent in the design and implementation of upward feedback programs, when there is misalignment between the goals of education leaders, teachers, and learners. While questions regarding how to better align systems and goals remain unanswered, it is clear that upward feedback mechanisms require an abundance of caution along with a coordinated effort and that targeting factors that mediate goal conflict offers a promising starting point.

## Additional Files

The additional files for this article can be found as follows:

10.5334/pme.818.s1 Appendix A. Interview Questions.

10.5334/pme.818.s2 Appendix B. Codebook (Codes and Operational Definitions).
