# Supplementary material for: Upward Feedback: Exploring Learner Perspectives on Giving Feedback to their Teachers
Source: Perspect Med Educ. 2023 Mar 22;2(1):99–108. doi: 10.5334/pme.818 (PMC10038106; doi:10.5334/pme.818)
Supplement: Appendix A. — Interview Questions. [file pme-12-1-818-s1.pdf]

Appendix A Interview Questions

Preamble (KW/KH): Thank you for your interest in this research study that explores your perspectives on teacher feedback. As a reminder, these conversations are confidential. We will only be saving the audio recording of this discussion; the video recording will be deleted. That said, you are welcome to turn your camera off anytime if you prefer. Encourage you to be candid - Your identity will only be known to me and KH/KW. Other members of the research team will only have access to de-identified transcripts. This interview should last about 45 minutes, and I will be asking you questions around how you tend to express value to your teachers, challenges you may face in offering feedback, and how you think feedback you provide is used. As we go along, please let me know if any questions require clarification. Do you have any questions before we begin?

Demographic Questions

1. In what year of medical school are you enrolled?
2. At what site are you studying

Explores how learners' express value to their teachers (note: probe whether answers differ for clinical vs. classroom settings)

3. Think about a teacher who has positively impacted you. Tell me a little about that experience. Did you do anything to show that you valued what they taught you? If so, what did you do and why did you feel compelled to do that? If not, why not?
    a. In other experiences, how have you tried to show that you valued the teaching provided by those who have positively impacted you?
    b. What determines whether or not you take steps to express your appreciation of the teaching you've received? What determines your particular strategy?
4. Think about a teacher who you felt could improve their teaching. Tell me a little about that experience. Did you do anything to try to address this? If so, what did you do and why did you feel compelled to do that? If not, why not?
    c. In other experiences, how have you tried to influence/improve your teachers' teaching style and approaches?
    d. What determines whether or not you take steps to try to improve the teaching you've received? What determines your particular strategy?
5. Think about a teacher who you felt taught "okay" or was average. Tell me a little about that experience. Did you do anything to try to show what you did or did not value? If so, what did you do and why did you feel compelled to do that? If not, why not?
    a. In other experiences of 'middle of the road' teachers, how have you tried to influence/improve your teachers' teaching style and approaches?
6. Have your perceptions toward teacher feedback changed over the course of your medical education?

Explores challenges or other interfering factors in offering feedback

7. What barriers (if any) prevent you from offering feedback to your teachers?
8. Have you ever felt like you could not be perfectly honest in giving feedback to teachers? What was the feedback you wanted to give, and what did you end up giving? Why were you reluctant to say what you wanted to?

    a. Is there a relationship between the type of feedback you’re providing and the level of safety you feel in providing that feedback?
9. While we're trying to understand how feedback about teaching is conceived from students' point of view both for the sake of incentivizing teachers and quality improvement, we appreciate that such things can't fall entirely on the shoulders of trainees. Do you have any thoughts regarding how medical programs could optimize the impact of the insights students have to offer without over-burdening them?

Explores students’ perceptions of whether their feedback is used/meaningful

10. What do you think is typically done with feedback students provide to teachers?
    a. What have you been told by the medical program or other sources about how feedback is used?
11. What are your views on how feedback is used by the teacher or the medical school, and whether they are meaningful? Do your views change depending on the type/nature of the feedback?
12. Have you ever observed changes in a teacher’s behaviour based on feedback you provided? If so, what changed and what do you think most enabled the change? If not, why not?
13. Do you think it likely that there are barriers to teachers adopting feedback from students?
    a. Can you think of ways in which the medical program could help reduce those barriers?

Closing

14. Do you have any other thoughts on the topic of providing feedback to your teachers that you haven’t yet had a chance to express?
