# Supplementary material for: Upward Feedback: Exploring Learner Perspectives on Giving Feedback to their Teachers
Source: Perspect Med Educ. 2023 Mar 22;2(1):99–108. doi: 10.5334/pme.818 (PMC10038106; doi:10.5334/pme.818)
Supplement: Appendix B. — Codebook (Codes and Operational Definitions). [file pme-12-1-818-s2.pdf]

Appendix B - Codebook (Codes and Operational Definitions)

I. Approaches to feedback

a. Formal Channels
   i. Teacher Assessment program (ie. One45)
   ii. Student mistreatment site
   iii. Student Affairs/DSSL/Program staff/leads
   iv. Award nominations
   v. Verbal

b. Informal channels
   vi. Verbal
   vii. Email
   viii. Card/Gift
   ix. Engagement in learning process
   x. Talking with peers/other cohorts
   xi. Other (e.g. going into same specialty, wanting to become a teacher themselves)

c. Content
   i. Behaviours
   ii. Impact
   iii. Generic comments
   iv. Positive
   v. Negative
   vi. Neutral
   vii. N/A (e.g. choosing not to give feedback)

II. Factors influencing decision making

a. Student factors
   i. Learner's state of mind
   - **Emotionality:** for some, feeling intense emotions can lead students to offer feedback immediately, but when emotions fade are less compelled to. Alternatively, some appreciate having time to calm down as it allows learner to gather their thoughts and provide feedback.
   - **Feedback burnout:** feeling like there are too many feedback forms lead to less investment into giving teachers feedback
   - **Intimidating**: feeling intimidated or scared to give feedback to someone in a senior position can influence decisions to give feedback
   - **Confidence about ability to give feedback:** the extent to which the learner feels they are able to offer useful feedback can influence their approaches
   - **Stage in medical school:** variances in feedback approaches due to level of the learner (e.g. in later years, student feels better equipped to judge the teachers receptivity to feedback)

   ii. Perceived influences and impacts on learner
   - **Impacts on career/education:** perceptions of whether giving feedback is going to influence their future, for better or worse (e.g. will give feedback regardless of teacher quality if it will get them a reference letter in return, or learner won't give any negative feedback for fear teacher will treat them even worse)
   - **Worried about teachers' perception of learner:** concern that giving feedback will taint the teachers view of the learner (e.g. that they'd prefer the teacher have an impression of them solely based on the teaching experience)

   iii. Learner networks
   - **Other students' experiences:** learners can talk to each other about their experiences and subsequent changes (if any) in teaching quality as a result of their feedback, and use those stories to inform their own approaches

b. Teacher Factors
   i. Teacher-focused

- **Quality of teaching/type of feedback (ie. positive, negative, neutral):** a leaner feeling more/less driven to offer feedback to a teacher depending on how good/bad the teacher was
- **Preceptor career impacts including recruitment/retention:** being more/less likely to offer feedback if the learner thinks it will impact the teachers position (e.g. not giving feedback because they know it'll create a bigger problem if that teacher is removed)

ii. Relationship-focused

- **Relationship factors:** approaching feedback differently, depending on the type of teacher-learner relationship (e.g. if there is trust there, if the relationship is longitudinal, etc.). Longitudinal relationships are a double-edged sword. If a good experience, there is more likely to be trust and learner may feel safer offering even constructive feedback. If negative though and needing to work with teacher again, less likely to offer feedback as higher chance of having repercussions.
- **Perceived Receptivity/openness:** making decisions on whether to offer feedback based on whether they think the teacher is receptive to what the learner has to say
- **Considerations about teachers' feelings:** not wanting to hurt the teachers' feelings (e.g. might be a bad teacher but the learner assumes they have good intentions)

c. Context

i. Considerations in the moment

- **Time:** feeling pressed for time can influence whether learners invest time and effort into giving feedback to teachers
- **Situational factors:** learners may feel comfortable giving feedback in one setting but not another (e.g. one-on-one may be more conducive to a feedback conversation than being in a room full of attendings).

ii. Culture & Environment

- **Feedback culture**: learners are influenced by the extent to which the medical school/site values and promotes learner feedback
- **Site Differences:** some learners will compare their approaches to feedback in one site vs. another. E.g smaller sites may have more perceived repercussions because learner more likely to work with preceptor again
- **Boundaries (real or perceived):** students can view some modalities of feedback as being inappropriate given the nature of the teacher-learner relationship. Buying them dinner for example may be appropriate in one context but not the other.

iii. Properties of Feedback Tools and Processes

- **Process/where feedback goes and its impact:** the learners' understanding/transparency of who sees feedback and what is done with it can influence their approaches (e.g. a learner who thinks nothing is done with their feedback will perceive it to be a waste of their time, or a learner who offered feedback and it didn't go well, is hesitant to do so in the future)
- **Whether/type of feedback and teaching given in return:** there can be a sense of reciprocity where the student will invest giving feedback to the teacher, to the extent that the teacher invested in feedback/teaching the learner
- **Anonymity:** Learners consider the extent to which their feedback is identifiable when giving feedback to teachers. Even if feedback is claimed to be 'anonymous', learners can be distrustful of this.
- **Modality:** learners consider which platform will convey message most meaningfully (e.g. will provide positive feedback f2f as it's more memorable, and negative feedback on one45 because it feels safer to be honest)

III. Perceived value and utility of upward feedback

a. Goals of providing feedback

i. Teacher-centric

- **Influencing teaching quality: trying to convey to teachers which behaviours they should** continue and which they should adapt.

- **Informing leadership/the medical school:** conveying to the program that certain teachers are effective or ineffective so they can institute rewards (e.g. awards) or consequences (e.g. removing them from teaching)
- **Encouraging/discouraging teachers:** wanting good teachers to feel good about their teaching, or ineffective teachers to reconsider their teaching involvement
- **Influencing teachers position/contract:** specifically trying to have a teacher removed or promoted
- Learner-centric
- **Helping other medical students:** trying to give future learners a better experience, or speaking up for those who feel they can't
- **Feeling good about self:** feeling a sense of accomplishment for speaking up

b. Perceptions of how feedback is used and applied:
- **Used and applied by teacher:** the extent to which the learner thinks the teacher reads and applies learner feedback, ranging from not reading it at all, to reading it and tossing it, to reading it and thinking about it, to internalizing and applying it to improve their teaching
- **Used and applied by leadership/program** : the extent to which the learner thinks that the medical school staff/leadership reads feedback and uses it to improve the quality of teaching for medical students (by influencing teacher contracts, for example)
- **Unknown:** expressing a lack of transparency or clarity on what is done with learner feedback

c. Perceived barriers to teachers adopting feedback

Factors preventing one from appreciating feedback
- **Generational differences:** teachers were taught in a different generation and therefore aren't equipped to change their ways
- **Power differential:** teachers aren't going to take feedback from novice learners seriously
- **Refusal to change/Ego:** teachers do not want to or care to change their teaching approaches because they have ego's
- **Cultural background**: some teachers find it difficult to adjust their communication styles or other approaches due to their cultural background (e.g. those who don't have English as their first language)
- **Don't agree with feedback:** teachers disagree with feedback given and brush it off

Prioritization
- **Time constraints**: teachers don't have enough time to read feedback and do the work to improve
- **Teaching not main job of doctor/not priority:** doctors first, teachers second and as such, are less invested in improving their teaching (e.g. teachers only asking for feedback because they have to, not because they want to)

Lack of knowledge on how to change
- **Not knowing how to change based on feedback given:** teachers do not have the appropriate resources to apply feedback, or can't interpret feedback because it's not delivered through a conversation
- Suggestions: suggestions to improve the likelihood and ability for teachers to improve their teaching based on learner feedback (e.g. reducing the number of forms, having someone from the program to be able to talk to about teachers, not inviting ineffective teachers back to teach)
